# Cancer epigenetics: from laboratory studies and clinical trials to precision medicine

**DOI:** 10.1038/s41420-024-01803-z

**Published:** 2024-01-15

**Authors:** Xinyang Yu, Hao Zhao, Ruiqi Wang, Yingyin Chen, Xumei Ouyang, Wenting Li, Yihao Sun, Anghui Peng

**Affiliations:** 1 Guangdong Provincial Key Laboratory of Tumor Interventional Diagnosis and Treatment, Zhuhai Institute of Translational Medicine, (Zhuhai People’s Hospital Zhuhai Clinical Medical College of Jinan University), Zhuhai, 519000 China; 2 Department of Spinal Surgery, Yichang Central People’s Hospital Affiliated with China Three Gorges University, Yichang, Hubei 443000 China; 3 Department of Pharmacy, Zhuhai People’s Hospital, Zhuhai People’s Hospital (Zhuhai Clinical Medical College of Jinan University), Zhuhai, Guangdong 519000 China

**Keywords:** Epigenetics, Cancer epigenetics

## Abstract

Epigenetic dysregulation is a common feature of a myriad of human diseases, particularly cancer. Defining the epigenetic defects associated with malignant tumors has become a focus of cancer research resulting in the gradual elucidation of cancer cell epigenetic regulation. In fact, most stages of tumor progression, including tumorigenesis, promotion, progression, and recurrence are accompanied by epigenetic alterations, some of which can be reversed by epigenetic drugs. The main objective of epigenetic therapy in the era of personalized precision medicine is to detect cancer biomarkers to improve risk assessment, diagnosis, and targeted treatment interventions. Rapid technological advancements streamlining the characterization of molecular epigenetic changes associated with cancers have propelled epigenetic drug research and development. This review summarizes the main mechanisms of epigenetic dysregulation and discusses past and present examples of epigenetic inhibitors in cancer diagnosis and treatment, with an emphasis on the development of epigenetic enzyme inhibitors or drugs. In the final part, the prospect of precise diagnosis and treatment is considered based on a better understanding of epigenetic abnormalities in cancer.

## Facts

- Epigenetic regulatory mechanisms involve cancer biology, especially DNA methylation, histone acetylation, and miRNAs.
- The expression of tumor-related genes is closely related to the epigenetic regulatory process of tumors.
- DNMTi, HDACis, BETis and other epigenetic therapies are constantly being updated and used in the clinic.
- Epigenetic combination therapy is a promising direction.
- Multi-omics, gene therapy, and AI are favorable transitions from epigenetic therapy to precision medicine.

## Open Questions

- Can the therapeutic efficacy of solid tumors be improved by combining therapies targeting different epigenetic markers?
- Which changes measured in epigenetic cancer precision diagnosis and treatment are temporary, and which are true tumor biomarkers?
- How to compare the results in personalized treatment when the conclusions of laboratory studies and clinical studies are contradictory?

## Introduction

Genomic DNA in eukaryotic cells is packaged around histones into a structure called the nucleosome, which further folds to produce the higher-order chromatin structure. Nucleosomes are the basic structural units of chromatin. They comprise of 146 bp of DNA wrapped in octamers of four core histones (H3, H4, H2A, and H2B dimers) [1]. The dynamic spatial organization of chromatin is critical for nucleosomes localization, the recruitment of transcriptional regulators, chromatin accessibility, and gene expression regulation. The compact spatial structure of nucleosomes exerts a universal inhibitory effect on mRNA transcription, whereas accessible spatial structure allows transcriptional regulators and RNA polymerases to access the DNA [2]. Changes to the chromatin structure are primarily regulated by epigenetic processes. Nucleosome remodeling involved in higher order folding of chromatin fibers responds to changes in epigenetic modifications, including DNA methylation, histone modification and RNA-mediated processes [3]. Modifications of histone tails (primarily in chromatin fibers) act as expression or repression markers through the acetylation and methylation of many different amino acids [4]. Chromatin can store and transmit epigenetic codes in the form of DNA methylation or post-translational histone modifications [5]. These modifications are inherited during cell replication; therefore, epigenetic dysregulation is a feature of nearly all human cancers. More specifically, regulatory factors (coupled with an irregular genome structure or abnormal gene expression) trigger the transformation of various normal cell and tissue types into malignances.

Cancer is a multifactorial disease caused by genetic variation, epigenetic dysregulation, and environmental factors [6]. Epigenetic modifications regulated by the three-dimensional (3D) organization of the genome are dynamic and reversible [7]. Therefore, epimutations reversal is the core application for small-molecule inhibitors. The emergence of next-generation sequencing (NGS) technology and artificial intelligence (AI) has advanced our understanding of epigenetic regulation in cancer. In fact, anticancer therapies targeting specific types of epigenetic mechanisms show potential in clinical trials, either as a single agent or in combination with other therapies.

## Mechanisms of epigenetic dysregulation in cancer

**Fig. 1:**
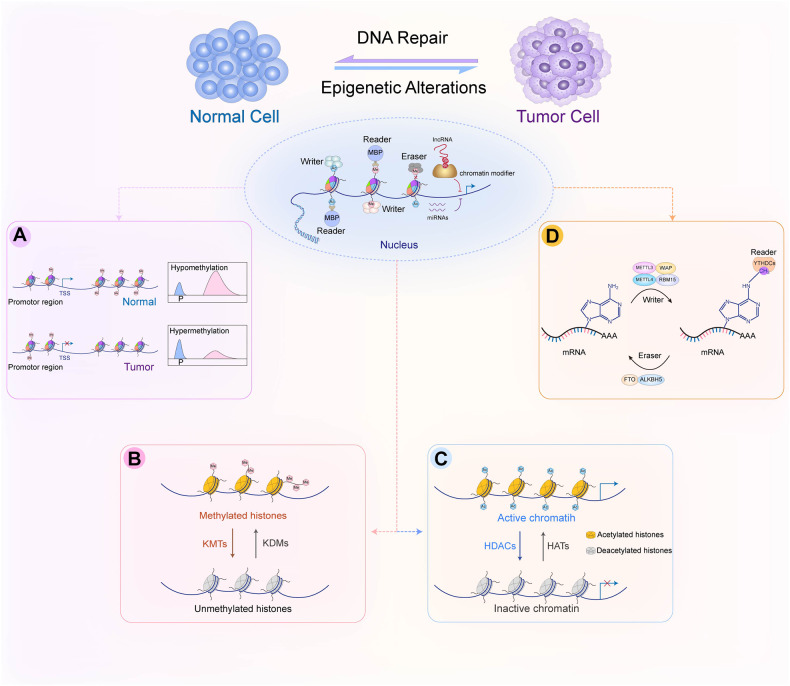
Epigenetic alterations associated with carcinogenesis. Epigenetic alterations involve DNA methylation, histone acetylation, and miRNA regulation that have reversible effects on gene silencing and activation through epigenetic enzymes and related proteins. Writers (DNMT, HAT, and KMT) are enzymes that add acetyl (Ac) and methyl (Me) tags to histones. MBDs are readers that recognize methyl-CpG and modify histones. Erasers (DNA demethylase, HDAC, and KDM) are responsible for removing chemical groups from DNA or histones. Noncoding RNAs (miRNAs and lncRNAs) are also involved in epigenetic regulation. **A** DNA methylation in normal and cancer cells. The overall hypomethylation and local hypermethylation of promoter regions are characteristics of cancer cells. P: promoter region. **B** Methylation and demethylation of lysine or arginine in histones. Lysine can be methylated once (me1), twice (me2) or three times (me3) catalyzed by KMT. Arginine is methylated once (me1) or twice (me2) catalyzed by KMT. These processes can be reversed by KDM. **C** HDAC removes acetyl groups from histone lysine residues. Acetylated histones are considered “active chromatin” allowing gene transcription, whereas deacetylated histones are “non-active chromatin” associated with gene silencing. **D** The methylation of m^6^A is installed by the RNA methyltransferase complex with the catalytic subunit METTL3/METTL4 (writer) and removed by demethylases, such as FTO and ALKBH5 (eraser). m^6^A reader proteins (YTHDCs) can specifically bind m^6^A transcripts. DNMT, DNA methyltransferase; HAT, histone acetyltransferase; HDAC, histone deacetylase; KDM, lysine demethylase; KMT, lysine methyltransferase; m^6^A, N^6^-Methyladenosine. MBP, methyl-CpG-binding domain protein.

Epigenetics is defined as a series of biological processes involving chromatin-mediated DNA template regulation, independent of changes in the original DNA sequence [8]. Protein complexes that control epigenetic modifications (including DNA methylation and covalent histone modification) can be divided into writers, readers, and erasers [9]. Epigenetic writers add distinct epigenetic chemical modifications to DNA or histones in the form of epigenetic markers. Readers are methyl-CpG-binding domain proteins (MBPs) that identify and interpret the specialized domains of modified proteins. Chromatin-modifying enzymes function as erasers by removing epigenetic markers (Fig. 1). Epigenetic dysregulation, including DNA, RNA methylation defects and abnormal post-translational modification processes, is commonly associated with all cancer types.

### DNA methylation

DNA methylation is characterized by the addition of a methyl group to the C5 position of cytosine residues by a DNA methyltransferase (DNMT). The resulting epigenetic methylation groups differentiate normal cells from cancerous and other diseased cells [10, 11]. The human genome encodes three conserved DNMT subtypes: DNMT1, DNMT3A, and DNMT3B [12]. DNMT1 is primarily involved in maintaining the pre-existing methylation pattern during DNA replication and the pattern during normal and cancer cell replication [13]. DNMT3 isoforms participate in de novo methylation and non-cytosine/guanine (CpG) methylation. Notably, DNMT3A and DNMT3B promote de novo DNA methylation of previously unmethylated sites to regulate the biological functions of embryonic development, cell differentiation, gene transcription, and cancer cell survival [14]. DNMT plays dual roles in tumor drug resistance. The mRNA expression levels of DNMT show diametrically opposed sensitivity to inhibitors in different tumors. For example, DNMT3B inhibition significantly increases the sensitivity of the inhibitor in pancreatic cancer [15], while ovarian cancers with high *DNMT* expression of are more sensitive to inhibitor treatment [16].

DNA methylation ensures the precise regulation of gene expression [17]. Hyper- and hypomethylation are relatively independent processes in the cancer genome and in tumor progression [18, 19]. Hypermethylated CpG islands in tumors are frequently located in gene promoter regions, in contrast to the overall hypomethylated regions. In fact, cancer cells are characterized by overall hypomethylation and local hypermethylation of promoters (Fig.1A). Hypermethylation of specific regions (such as tumor suppressor gene CpG islands) is associated with a myriad of carcinogenesis, including breast cancer [20], liver cancer [21], prostate cancer [22], and small-cell bladder cancer [23]. Methylation is involved in many physiological and pathological processes associated with transcriptional disorders in cancer and promoter hypermethylation is considered the primary mechanism of gene inactivation. Abnormal hypomethylation outside of CpG islands contributes to the increased expression of oncogenes [24, 25]. Mutation and inactivation of tumor suppressor genes can lead to DNA damage or uncontrolled cell growth, thereby promoting cancer progression [26]. Additionally, the potential reversibility of methyltransferase activity makes it an attractive target for therapeutic interventions, unlike genetic changes.

### Histone methylations

Histone methylation is a key determinant of complex chromatin state and is mainly regulated by lysine methyltransferase (KMT) and lysine demethylase (KDM). KMT and KDM play the roles of writers and erasers, respectively, in epigenetic regulation [27]. The canonical lysine methylation sites in humans are found at K4, K9, K27, K36, K56, and K79 on histone H3, and at lysine K20 on histone H4 [28]. The addition of methyl groups in the histone tails of arginine and lysine residues may involve monomethylation (me1), demethylation (me2), and trimethylation (me3) [29] (Fig. 1B). KMT and KDM are highly specific to the lysine residues in the substrate and the degree of methylation.

The KMT family uses S-adenosyl-L-methionine as a methyl donor to catalyze various lysine methylation events that modify core histones [30]. KMT2D is a KMT that increases the expression of the tumor suppressor gene (*PER2*) by positively regulating super-enhancers [31]. Polycomb group protein EZH2 represents another KMT that is highly mutated in many types of tumors and affects the expression of downstream target genes by the trimethylation of Lys-27 in H3 (H3K27me3). Hence, EZH2 is involved in a wide range of tumor processes [including tumorigenesis, cell cycle progression, metastasis, cancer immunity, and apoptosis [32]] and is a promising therapeutic target. Disruptor of telomeric silencing 1-like (DOT1L) is a histone 3 Lysine-79 (H3K79) methyltransferase that regulates transcriptional activation and elongation in mixed lineage leukemia (MLL) [33]. It involved in stem/progenitor cell regulation in solid tumors [34]. H3K9-methyltransferase SET domain bifurcated 1 (SETDB1) has a broad inhibitory effect mainly in open genomic compartments and is upregulated in various tumors [35]. Additionally, the euchromatin histone lysine methyltransferase (EHMT or G9a) family (including EHMT1 and EHMT2) primarily mediates H3 Lysine 9 dimethylation (H3K9me2) and plays important biological roles in cancers [36].

The KDM family utilizes the Jumonji-C (JmjC) domain to catalyze demethylation through oxidation of the methyl group [30]. The JmjC family of KDMs (JMJC-KDMs) plays important roles in the control of gene expression and chromatin structure [37]. For example, Lysine-specific demethylase 1 (LSD1 or KDM1A) is a member of the JmjC family that specifically demethylates histone lysine residues H3K4me1/2 and H3K9me1/2. In fact, it can interfere with the T-cell response of melanoma [38] and gastric cancer [39]. KDMs such as KDM1B and KDM6B has been reported to be able to promote cancer cell immune evasion, making them a potential therapeutic target [40, 41]. KDM1B inhibition can prevent the expansion of cancer stem cells induced by IFN-I [40]; KDM6B deletion can enhance a series of immune pro-inflammatory pathways such as interferon response, antigen presentation and phagocytosis in the glioblastoma (GBM) tumor immune microenvironment, indicating that KDM6B inhibition can overcome myeloid-derived immune suppression and enhance response to immunotherapy in GBM [41].

### Histone acetylation

Post-translational modifications of histones (including acetylation, methylation, phosphorylation, and ubiquitylation) play important roles in the epigenetic regulation of gene transcription. Histone acetylation and deacetylation are the most typical epigenetic post-translational modifications that occur in the NH_2_ terminal tail of core histones [42]. Two competing families of enzymes [histone lysine acetyltransferases (HATs) and histone deacetylases (HDACs)] regulate histone acetylation. More specifically, HATs catalyze the transfer of acetyl groups from acetyl-CoA to the amino group of histone lysine residues. This acetylation of histone tails promotes chromatin accessibility that facilitates positive transcription. Meanwhile, HDACs remove acetyl groups from the ɛ-amino lysine residues in histone tails, and effectively reduce access to transcription factors by forming a closed chromatin conformation to alter the transcription of oncogenes and tumor suppressor genes [43] (Fig. 1C).

There are 18 known human HDACs that deacetylate lysine substrates through zinc-binding or NAD^+^-dependent substrates [44]. These enzymes are further categorized into four classes. Class I HDACs are components of multiple inhibitory complexes (including HDAC1, HDAC2, HDAC3, and HDAC8) that are primarily localized in the nucleus and are generally expressed in the human genome. Class II HDACs are in the nucleus and cytoplasm and exhibit tissue-specific expression patterns and non-histone deacetylation activity. They are further divided into two subclasses, IIa (HDAC4, 5, 7, and 9) and IIb (HDAC6 and 10) [44]. Class III HDACs are comprised of SIR2-like proteins (including SIRT1, SIRT2, SIRT3, SIRT4, SIRT5, SIRT6, and SIRT7) that are involved in regulating multiple cellular processes such as survival, aging, stress response, and metabolism [43]. Class IV HDACs only comprise HDAC11, which shares partial homology with class I and II HDACs and acts as a long-chain fatty acid deacylase [45]. Class I, II, and IV HDACs rely on zinc-binding substrates and catalytic deacetylation, whereas Class III SIR2-like proteins use NAD^+^ as a reactant to deacetylate the acetyl acid residues of protein substrates [46].

Dysregulation of HDAC is often observed in various cancers and in many different stages of cancer, including (but not limited to) differentiation [47], cell cycle [48], angiogenesis [49], apoptosis [50], and autophagy [50]. Notably, HDAC inhibition downregulates the expression of apoptosis-related proteins. Therefore, HDACs can be used as a therapeutic target for abnormal cell growth and proliferation in cancer.

### RNA epigenetics

RNA modification is a prominent field of epitranscriptomics [51]. Dynamic RNA modifications represent a new level of control over genetic information. They can be selectively deposited to a set of transcripts by selective transcription factors and facilitate the coordinated utilization and turnover of the transcriptome. This is a fundamental mechanism for regulating the cellular transcriptome during development [52]. RNA methylation is an important process in epigenetics. N^6^-Methyladenosine (m^6^A) is the most abundant internal RNA methylation modification that accelerate pre-mRNA processing and mRNA transport to affect mRNA stability, splicing, and translation in mammalian cells [51]. The loss of METTL3 or METTL14, as key components of the RNA methyltransferase complex, significantly promotes the growth, self-renewal, and tumorigenesis of the human GBM stem cell [53]. Inhibition of the fat mass and obesity-associated protein (FTO, a m^6^A demethylase), can reduce oncogene-mediated cell transformation in leukemia [54]. Similarly, decreased expression of ALKBH5 (another m^6^A demethylase) reduce the percentage of breast cancer stem cells, thereby reducing the possibility of tumorigenesis [55]. Collectively, RNA-modifying enzymes that regulate m^6^A are writers (including METTL3 and METTL14), readers (YTHDCs), and erasers (FTO or ALKBH5) in various cancers (Fig. 1D).

There is growing evidence that not only protein coding associated RNAs (mRNA, tRNA and rRNA), but also non-coding RNAs (ncRNAs), such as microRNAs (miRNAs) and long non-coding RNAs (lncRNAs), have direct functional effects on gene expression. LncRNAs are defined as RNAs longer than 200 nucleotides and may regulate gene expression at multiple levels. They can regulate the structure and function of chromatin by interacting with DNA, RNA, and proteins to form multiple hybrids. The cis- and trans-regulation at the transcriptional and post-transcriptional levels coordinates the regulation of nuclear localization and function of lncRNAs, affecting the chromatin state, and ultimately regulates gene states at the proximal and distal ends [56]. LncRNAs can act as epigenetic drivers by mediating the recruitment of chromatin regulators at specific chromatin loci, and the specific regulatory pattern of functional lncRNAs may be used as cancer biomarkers and therapeutic targets. For instance, HOTAIR is a lncRNA that is overexpressed in several epithelial cancers. It triggers H3K27me3-mediated repression of *HNF1α* and *HNF4α* genes closely related to repression of the epithelial-mesenchymal transition (EMT) by recruiting the chromatin modifier, EZH2 [57]. It can also recruit *PRC2* to methylate histones on the *CDKN2B*/*P15-INK4b* promoter to facilitate transcriptional inhibition [58]. An oncopeptide encoded by an uncharacterized lncRNA (LINC00266-1) sensitizes m^6^A recognition and enhances the recruitment of RNA stabilizers to increase the stability and expression level of *c-Myc*, which promotes oncogenesis in colorectal cancer (CRC) [59]. miRNAs are highly conserved endogenous small (~22 nt) ncRNAs that participate in the epigenetic regulation of tumors as critical regulatory molecules. Promoter methylation or histone acetylation can abnormally regulate miRNA expression in cancer [60]. For example, the miR-29 family can directly target DNMT3A and DNMT3B [61]. Moreover, miRNAs are directly associated with the epigenetic mechanisms of their enzyme components through regulatory loops, thereby affecting the expression of a wide range of regulatory factors [62]. Alterations in miRNAs are the result of tumorigenesis and actively contribute to cancer development. miRNA expression profiles are better options for predicting cancer type and stage compared to mRNA expression profiles; therefore miRNAs are proposed as useful tools for cancer diagnosis or prognosis [63]. CpG hypermethylation suppresses miR-9-1 in breast cancer and miR-124a in colorectal tumors relative to normal tissues [64, 65]. Epigenetic silencing of miRNAs may reflect tissue specificity. Tissue- and cell-type-specific expression of miRNAs widely affect cell differentiation, cycling, aging, and metabolism [66]. Hence, the analysis of miRNA expression in tumor tissues or liquid biopsies can help direct cancer diagnosis, predict patient prognosis, and identify potential therapeutic targets.

## Epigenetic anticancer strategies

Different cells in tumor tissues exhibit variable epigenetic modification patterns throughout the genome or at individual genes; this suggests that there is epigenetic heterogeneity at the cellular level [67]. The reversibility of abnormal DNA methylation and acetylation patterns is a common target of cancer treatment. Many small-molecule inhibitors targeting chromatin- and histone-modifying enzymes to reverse epigenetic alterations in tumors and restore the normal epigenetic state are successful as cancer therapeutics in clinical trials (Table 1). The therapeutic effect is variable in solid tumors owing to the lack of biomarker-driven targeted therapies, which leads to a highly heterogeneous response. The clinical benefits of epigenetic monotherapy remain unknown; however, our understanding of tumor epigenetic regulation has expanded, leading to progress in the analysis of epigenetic therapies in clinical trials.

**Table 1:** Epigenetic inhibitors used in malignancies.

Target	Inhibitor	Associated cancer	Clinical status	Reference
DNMT	Azacitidine	Therapy-related myeloid neoplasms	Achieves disease response	[71]
	Decitabine	AML	Achieves disease response and better overall survival	[72]
	SGI-110	AML	Achieves disease response	[73]
	CP-4200	AML	Causes efficient reactivation of epigenetically silenced tumor suppressor genes	[74]
	MG98	Renal cell carcinoma	Inhibits the proliferation of growing cancer cells	[75]
	Nanaomycin A	Hepatocellular carcinoma	Exhibits antiproliferative effects	[76]
HDAC	Vorinostat (SAHA)	T-cell lymphoma	Determines complete and partial response rates	[80]
	Romidepsin (depsipeptide)	CTCL	Achieves disease response	[81]
	Belinostat	Peripheral T-cell lymphoma	Achieves disease response	[82]
	Panobinostat	MM	Achieves disease response	[83]
	TSA	Breast cancer	Has greater specificity for cancer vs normal cells	[84]
	Tubacin	Melanoma, CRC	Increases the extracellular release of a cancer stem cell marker	[85]
	MC1568, MC1575	Breast cancer, melanoma	Exhibits antiproliferative effects	[86, 87]
	Ricolinostat (ACY-1215)	GBM	Inhibits tumor cell growth	[88]
	IN-2001	Breast cancer	Suppresses tumor growth	[89]
	AR-42	Meningioma	Increases proapoptotic gene expression and decreases anti-apoptotic protein levels	[90]
	Givinostat (ITF2357)	BCP-ALL	Inhibits the proliferation and induces apoptosis	[91]
BET	JQ1	MM, AML, DLBCL, prostate cancer, breast cancer	Produces a potent antiproliferative effect associated with cell-cycle arrest and cellular senescence, terminal myeloid differentiation, and elimination of leukemia stem cells	[94–98]
	OTX015 (MK-8628)	B-cell lymphoma, neuroblastoma	Inhibits the proliferation of cancer cells; downregulates *c-Myc*, MYCN, and other oncogenes associated with super-enhancers	[99, 100]
	MS645	Triple-negative breast cancer	Inhibits cancer cell proliferation	[101]
	ABBV-075	AML, non-Hodgkin lymphoma, MM	Triggers apoptosis	[102]
	ABBV-744	Prostate cancer	Displaces BRD4 from AR-containing super-enhancers and Inhibits AR-dependent transcription	[103]
	I-BET151	GBM	Inhibits GBM cell proliferation	[105]
	CC-90011	Solid tumors	Achieves complete response or partial response; prolongs stable disease	[104]
	I-BET 762	Pancreatic cancer	Hinders multiple pathways associated with cell growth	[107]
KDM	ORY-1001	AML	Reduces the growth of cancer cells	[108]
KMT	BIX-01294	DLBCL, neuroblastoma	Inhibits cell proliferation and induces apoptosis of cancer cells	[109, 110]
	UNC0638	non-SCLC	Inhibits cell growth and induces apoptosis	[111]
	Pinometostat	MLL	Inhibits the proliferation of leukemia cell lines harboring MLL-r and induced sustained regressions	[112]
	EPZ004777	MLL	Selective kills cells bearing the MLL gene translocation	[113]
	GSK126	Myeloid-derived suppressor cells	Inhibits the growth of tumor cells	[114]

*AML* acute myeloid leukemia, *AR* androgen receptor, *BCP-ALL* B-cell precursor acute lymphoblastic leukemia, *BET* bromodomain and extraterminal domain, *CTCL* cutaneous T-cell lymphoma, *CRC* colorectal carcinoma, *DLBCL* diffuse large B-cell lymphoma, *DNMT* DNA methyltransferase, *GBM* glioblastoma, *HDAC* histone deacetylase, *KDM* lysine demethylase, *KMT* lysine methyltransferase, *MM* multiple myeloma, *MLL* mixed lineage leukemia, *SCL*C small cell lung cancer.

### DNMT Inhibitors

Aberrant inherited DNA methylation and gene silencing accumulates in cancer cells. Pharmacological inhibitors of DNA methylation were developed to reverse epigenetic changes via DNMT inhibition. Azacitidine (AZA) and decitabine (DAC) are the first clinically used DNMT inhibitors (DNMTis) that have been approved by the FDA to treat myelodysplastic syndromes (MDS) [68, 69]. These two azanucleosides contain a nitrogen at the C-5 position of their pyrimidine ring that blocks the catalytic activity of DNMTs, resulting in DNMT1 degradation and genome-wide DNA hypomethylation, and facilitating the re-expression of tumor previously silenced by DNA methylation [70]. These inhibitors lead to the preferential upregulation of genes with promoter DNA methylation and exhibit high targeting selectivity in cancer cells.

AZA is a safe and effective treatment option for patients with therapy-related myeloid neoplasms [71]. Meanwhile, DAC performs well in first-line and salvage therapy for patients with acute myeloid leukemia (AML) that have a poor prognosis and are not candidates for intensive chemotherapy [72]. Additionally, CP-4200 and SGI-110 are respective analogs of AZA and DAC that exhibit potential DNMT inhibitory activity in AML [73, 74]. MG98 is a promising human DNMT1 antisense inhibitor that does not require incorporation into DNA since it downregulates *DNMT1* expression and cancer cell proliferation by reducing the cellular mRNA concentration in a dose-dependent manner in renal cell carcinoma [75]. Moreover, nanaomycin A has a selective inhibitory effect on DNMT3B and antiproliferative effects in hepatocellular carcinoma [76]. The antitumor efficacy and characteristics of these inhibitors define DNMT drivers and potential targets in cancer.

### HDAC inhibitors

Cancer often exhibits abnormal acetylation modifications that can lead to the silencing of key tumor suppressor genes [77]. HDAC inhibitors (HDACis) can block HDAC deacetylase activity. This restores cellular acetylation homeostasis and results in unrestricted HAT activity. This leads to increased gene transcription and ultimately triggers a series of biological responses to hinder tumor cell growth or survival, including chromatin remodeling, tumor suppressor gene transcription, growth inhibition, and apoptosis. In particular, the main mechanism of HDACis is the activation of intrinsic apoptosis pathways [78]. HDACis selectively target tumor cells in preclinical studies. This results in the approval of several drugs to treat certain hematologic malignancies.

HDACis highlight treatment stratification in solid tumors. For example, non-*YAP1*-driven small cell lung cancer (SCLC) and brain tumors with *IDH1/2* mutations represent cancer subsets that are suitable for HDAC-targeted therapy [79]. HDACis are divided into selective and nonselective inhibitors according to target selection. The most widely studied and commonly used are non-selective HDACis. Vorinostat (SAHA) and romidepsin (depsipeptide) have been approved by the FDA for the treatment of cutaneous T-cell lymphoma (CTCL) [80, 81], whereas belinostat (Beleodaq/PXD101) and panobinostat (LBH-589) have been approved for the treatment of peripheral T-cell lymphoma and multiple myeloma (MM), respectively [82, 83]. The emergence of selective HDACis provides a reliable tool to resolve the function of HDAC subtypes, and a safer and more effective option than broad-spectrum HDACis. Trichostatin A (TSA) is an inhibitor of class I and II HDACs with noncompetitive effects and potent dose-dependent antitumor activity against breast cancer [84]. Notably, tubacin is a specific selective HDACi of class IIb that can increase the extracellular release of CD133^+^ extracellular vesicles (a cancer stem cell marker) in human FEMX-I metastatic melanoma and Caco-2 colorectal carcinoma cells [85]. MC1568 and MC1575 are derivatives of arylyl-pyrrole-hydroxyamide that are selective for class IIa HDAC and HDAC6, respectively, and exhibit antiproliferative effects in estrogen receptor-positive breast cancer cells [86] and human melanoma cells [87]. Moreover, the HDAC6-selective inhibitor ricolinostat (ACY-1215) significantly inhibits GBM cell growth [88]. Meanwhile, newly developed HDACis shows promising prospects for tumor diagnosis and treatment. For example, IN-2001 exhibits potential antitumor activity in human breast cancer cells [89], whereas AR-42 demonstrates potential in the diagnosis and treatment of meningiomas. Moreover, givinostat (ITF2357) exerts antitumor activity and selectively kills cancer cells in B-cell precursor acute lymphoblastic leukemia [90, 91]. Collectively, HDACis show considerable value in the diagnosis and treatment of various cancers.

### BET family inhibitors

Bromodomain and extraterminal domain (BET) proteins, including BRD2, BRD3, BRD4, and BRDT can recognize lysine acetylation that is closely associated with DNA replication, DNA damage repair, chromosomal remodeling, and oncogene transcription. Each BET family member contains two tandem N-terminal bromodomains (BD1 and BD2) and an extra C-terminal domain that functions as a key epigenetic reader of oncogenic networks in various cancers [92]. BRD4 is considered a universal transcriptional regulator with enrichment on super-enhancers that drive the expression of cancer-specific genes [93]. Meanwhile, BET inhibitors are a new generation of selective anticancer drug that interfere with transcriptional initiation and elongation by blocking BET functions. BET inhibitors displace BRD4 from the regulatory region to inhibit gene expression, and the antitumor efficacy of BET family inhibitors is particularly promising for cancers with increased expression of oncogenic transcription factors, such as *c-Myc*.

BRD4 contributes to the maintenance of *c-Myc* expression to promote the abnormal self-renewal of AML cells [94]. Meanwhile, JQ1 is a prototype BRD4 inhibitor with proven therapeutic benefits in MM [94], AML [95], diffuse large B-cell lymphoma (DLBCL) [96], prostate cancer [97], and breast cancer [98]. OTX015 (MK-8628) is a first-in-class BRD2/3/4 inhibitor that inhibits cell proliferation in hematological malignancies and neuroblastoma, and downregulates the expression of *c-Myc*, *MYCN*, and other oncogenes associated with super-enhancers [99, 100]. Furthermore, MS645 is a bivalent BRD4 inhibitor that suppresses the proliferation of triple-negative breast cancer cells by blocking the binding of BRD4 to MED1 and YY1 transcription factors [101]. ABBV-075 is a novel BET inhibitor that triggers apoptosis in AML cells, non-Hodgkin lymphoma, and MM cells [102], whereas ABBV-744 selectively targets the BD2 domain, displaces BRD4 from androgen receptor (AR)-containing super-enhancers, and inhibits AR-dependent transcription. This shows better antitumor activity than ABBV-075 in a mouse xenograft model using human prostate cancer cells [103]. CC-90010 is another next-generation BET inhibitor with encouraging antitumor activity in patients with advanced solid tumors [104]. The abundances of BRD2 and BRD4 are significantly increased in GBM. Therefore, treatment with BET protein inhibitor (I-BET151) inhibits GBM cell proliferation [105]. Moreover, OTX015 exhibits a higher antiproliferative effect than its analog (JQ1) in GBM cell lines [106]. Meanwhile, JQ1 and I-BET 762 effectively interfere with multiple pathways associated with cell growth in pancreatic cancer [107]. Overall, the traditional small-molecule BRD4 inhibitor JQ1 and the newly developed BET inhibitors show promising results in various human cancers.

### KMT & KDM inhibitors

The past decade has seen tremendous progress in the characterization of the regulation of methyl modifications by KMTs and KDMs. Inhibitors of these two enzymes have also attracted much attention in the treatment of cancer. For instance, the effect of a LSD1/KDM1A selective inhibitor (ORY-1001) was evaluated in patients with blood disorders [104, 108]. BIX-01294 is a small-molecule inhibitor of EHMT2 that inhibits cell proliferation and induces apoptosis of cancer cells in DLBCL [109] and human neuroblastoma [110]. Similarly, UNC0638 is a EHMT2 inhibitor that substantially reduces cell growth and induces apoptosis in non-SCLC cells [111]. The involvement of DOT1L in multiple cancer processes provides compelling support for its inhibition as a basis for targeted therapeutics against cancers. Pinometostat (EPZ-5676) is a first-in-class small-molecule DOT1L inhibitor, for the treatment of adult acute leukemia [112]. EPZ004777 is another potent selective DOT1L inhibitor that selectively kills cells carrying MLL-associated gene translocation and prolongs survival in vitro [113]. Treatment with GSK126 (an EZH2 inhibitor) inhibits the growth of immune-deficient tumor cells [114]. In addition, small-molecule EZH2 inhibitors eliminate tumor cell growth in diffuse intrinsic pontine glioma through a mechanism induced by the tumor suppressor protein, p16^INK4A^ [115]. These studies and clinical trials suggest that KDMs and KMTs inhibitors are prospective for cancer clinical diagnosis and treatment.

### Combination therapy strategies

The two main challenges associated with epigenetic mono-treatment are vulnerability to resistance and limited activity. This may be solved by combined therapies targeting different epigenetic markers. Most combination therapy clinical trials are ongoing, while some show encouraging results (Table 2).

**Table 2:** Combination therapeutics used in malignancies.

Combination forms	Combination therapeutics	Associated cancer	Advantages	Reference
DNMTi+HDACi	azacytidine + vorinostat	MDS; CMML	more effective than monotherapy	[116]
	azacytidine + entinostat	CRC	improved antitumor activity	[117]
Epi-drug + targeted drug	ACY-1215 + bortezomib	MM	delayed tumor growth; prolonged survival	[121]
	TSA + palladium nanoparticles	cervical cancer	increased the potential for successful treatment	[122]
	JQ1 + γ-secretase inhibitors	T-ALL	countered the resistance of γ-secretase inhibitors	[123]
Epi-drug + immunomodulators	Panobinostat + bortezomib+dexamethasone	MM	improved progression-free survival	[126]
	PD-1 blockers +Decitabine	CRC	inhibited tumor growth; prolonged survival	[127]
	Azacytidine+pembrolizumab	MDS	safe with controllable toxicity	[128]

*CMML* chronic myelomonocytic leukemia, *CRC* colorectal carcinoma, *MDS* myelodysplastic syndromes, *MM* multiple myeloma, *T-ALL* T-cell acute lymphoblastic leukemia.

DNMTis are highly selective for cancer-related genes, while certain HDAC inhibitors are nonselective. This suggests that combining these inhibitors will provide better therapeutic efficacy. Combinatorial therapy comprising vorinostat and AZA is more effective than monotherapy in MDS and chronic myelomonocytic leukemia (CMML) [116]. Meanwhile, combining DAC with KDM1A, EHMT2, or EZH2 inhibitors synergistically increases gene regulation while maintaining DAC selectivity compared to the combination of DAC with HDACi [70]. Moreover, the combined use of AZA with the HDACi, entinostat, significantly improves the antitumor activity of checkpoint inhibitors in computed tomography 26 (CT26) mice, a model of mismatch repair-proficient CRC [117]. Additionally, the combination of DNMTi and HDACi exerts a synergistic activating effect on silent tumor suppressor gene expression [118, 119]. Combination therapy of DNMTi and HDACi simultaneously induces the expression of major tumor suppressor genes in MM cells and inhibits the expression of key oncogenes, such as *MYC* and *IRF4* [120]. Therefore, the combination of two inhibitors synergistically induces gene expression while maintaining selectivity to increase the likelihood of targeting specific tumor types based on gene expression profiles.

The combination of epigenetic therapy with other cancer treatments represents an effective therapeutic approach. Various epigenetic agents were combined with targeted drugs. For example, DNMTis can be combined with inhibitors targeting histone methylation to achieve synergistic effects that preferentially target cancer-associated genes [70]. ACY-1215 is a selective HDAC6 inhibitor showing promising preclinical interactions with bortezomib in MM [121]. Moreover, the combination of TSA and palladium nanoparticles synergistically increases the potential for successful treatment of cervical cancer [122]. Meanwhile, the combination of JQ1 and γ-secretase inhibitors in the treatment of T-cell acute lymphoblastic leukemia (T-ALL) counter the resistance to targeted therapy with γ-secretase inhibitors [123].

Epigenetic therapies can improve the efficacy of immune checkpoint therapies. Notably, epigenetic therapy may sensitize patients to the reversal of immune tolerance, while epigenetic and immunomodulatory combination therapy results in tumor DNA demethylation, increased RNA transcription, and immunomodulation [124]. In fact, DNMTis alter the expression patterns of genes associated with innate and adaptive immunity, and immune evasion in tumor tissues [125]. DNMTis also stimulate immune responses against cancers, resulting in their sensitization to immunotherapy. The combination of panobinostat (HDACi) with bortezomib (a proteasome inhibitor) and dexamethasone (an immunomodulatory drug) improves progression-free survival in patients with MM and is approved by the FDA [126]. Moreover, treatment with a combination of PD-1 blockers and DAC results in more significant tumor growth inhibition and prolonged survival in CT26 mice with CRC [127]. A phase II trial demonstrates that AZA and pembrolizumab elicit antitumor activity in some patients with MDS. Importantly, this combination therapy is relatively safe with controllable toxicity [128].

The approach of multi-target combination construction enhances the antitumor effect by simultaneously activating multiple anticancer pathways and is a potential alternative to combination therapy. Multi-target hybrid inhibitors concomitantly regulating two or more targets and inhibiting biochemically related targets improve the treatment response and drug resistance of tumor patients. C02S is a dual DNMT and HDAC inhibitor hybrid compound that exhibits significant enzymatic inhibitory activity against DNMT1, DNMT3A, DNMT3B and HDAC1. It induces the expression of *p16*, *p21*, and *TIMP3* and causes DNA damage, while modulating multiple cancer markers and exerting tumor growth suppression in a mouse model of breast cancer [129]. A dual LSD1/HDAC co-inhibitor called corin has marked antiproliferative activity in melanoma cell lines and cutaneous squamous cell carcinoma cell lines [130]. Double hybrid molecule 5, targeting HDAC and EZH2 inhibits proliferation and kills cancer cells at low micromolar concentrations in several cancer cell types [131]. The epigenetic pharmacological strategies of drug mixtures acting on different biological pathways are emerging as a new approach towards modern cancer therapy.

## Perspectives in precision medicine

“Precision medicine” is tailored to the individual medical or subgroup characteristics of each patient to achieve the highest possible therapeutic effect and minimize toxicity and side effects [132]. Notably, patients with the same gene variant can respond differently to treatments using the same drugs. Genetic and epigenetic diagnostic testing are required to establish the potential of personalized medicine. Epigenetic modifications and drug responses are mutually regulated. Thus, addressing the compatibility between epigenetic research and clinical applications is a major challenge in precision medicine.

**Fig. 2:**
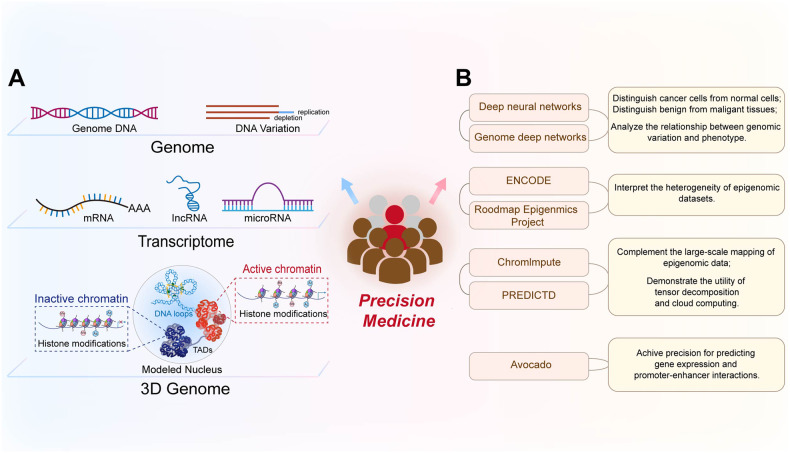
Approaches contributing to personalized medicine. **A** Multi-omics comprehensively analyzes the genome, transcriptome, and epigenetic panels of tumors from multiple perspectives. **B** Classification diagram of AI application in precision medicine.

Multi-omics and AI have highlighted the critical role of epigenetic mechanisms in cancer. They provide new opportunities to identify tumor epigenetic biomarkers for early screening, diagnosis, and the development of personalized treatment (Fig. 2).

The unique epigenetic pattern of chromatin drives cell-type-specific expression. Advances in NGS technologies facilitate the identification of complex epigenetic patterns specific to cancer cells and provide important insights to guide the identification of new therapeutic targets and predictive biomarkers. Moreover, genetic and epigenetic data, (including structural variations, gene expression profiles, DNA methylation patterns, histone modification profiles, and 3D structures of the cancer genome) are key factors in personalized medicine (Fig. 2A). Multi-omics technologies that can profile genomics, transcriptomics, and 3D genomics and elucidate the interactions between genetic and epigenetic changes in cancer biology, are effective in precise disease management and prediction. Whole-genome and whole-exome sequencing can trace genomic variations in different tumors [133]. Meanwhile, RNA-seq can capture microarray transcription profiles and dissect tumor heterogeneity [134]. Currently, whole-genome bisulfite sequencing is the gold standard method for analyzing DNA methylation data on a genome-wide scale at single-base resolution [135]. Additionally, chromatin accessibility affects the DNA binding to transcription factors and regulatory elements. This provides important insights into the mechanisms by which cancer genomes are activated and silenced [136]. The assay for transposase-accessible chromatin using sequencing (ATAC-seq) can assess the chromatin accessibility landscape of primary human cancers [137]. Meanwhile, interference with the 3D structure of the genome can lead to ectopic oncogene activation by interacting with proximal or distant enhancers and promoter regions that initiate oncogene transcription. Thus, chromatin immunoprecipitation and sequencing (ChIP-seq) technology can be employed to identify activity enhancers and super-enhancers on a genome-wide scale based on H3K4me1 and H3K27ac histone markers, respectively [138]. Additionally, high-throughput genome-wide chromatin spatial capture (HiC) analysis can identify abnormal enhancer-promoter interactions throughout the tumor genome [139]. The rapid development of these technologies is conducive to defining the epigenomic panorama of cancer and facilitates further integration of epigenomic indicators in clinical applications. Indeed, the combination of epigenetic techniques with more commonly used platforms (such as whole-genome sequencing and RNA-seq) will provide comprehensive insights regarding genomic and epigenomic abnormalities in patients with cancer. Additionally, the application of multi-omics approaches to identify diagnostic biomarkers will improve therapeutic interventions by providing data for personalized treatment.

AI is a novel medical tool that includes machine and deep learning; its application significantly improves cancer-associated precision medicine (Fig. 2B). More specifically, AI can automate the initial image decoding process, quantify stained tumor slice images or radiological image features, accurately distinguish cancer cells from non-cancer cells, and identify the specific tumor shape, size, cancer subtype, and lesion spread [140]. Computer-aided testing enables the systematic processing of tumor features (such as the detection of clustered microcalcifications in screening mammography) as an indicator of early-stage breast cancer [141, 142]. Deep neural networks are powerful algorithms with high precision in digital image processing to distinguish cancer cells from normal cells, and benign tissues from malignant tissues [143, 144]. Recently, the emerging field of “imaging genomics” has correlated radiographic features with biological data, including somatic mutations, gene expression, and chromosome copy numbers, among other molecular features. In particular, the deep neural network-based method of genome deep learning applies deep neural networks to genome point mutations to effectively analyze the relationship between genomic variation and phenotype [145]. Large consortia [including the Encyclopedia of DNA Elements (ENCODE) and the Roadmap Epigenomics Project] can interpret a heterogeneous collection of thousands of epigenomic datasets in the field of epigenetics. In fact, numerous analyses were performed on different human cell types, with thousands of epigenomic measurements performed on every base pair in the human genome [146]. Subsequently, the ChromImpute software and the PaRallel Epigenomics Data Imputation with Cloud-based Tensor Decomposition (PREDICTD) tool were combined to complement the large-scale mapping of epigenomic data and demonstrate the utility of tensor decomposition and cloud computing [147, 148]. Schreiber et al. proposed the multi-scale machine learning model, Avocado, which is a combination of tensor factorization and deep neural networks that compresses epigenomic data into dense, information-rich representations [149]. Avocado outperforms models trained directly on epigenomic data in a variety of genomic tasks and achieves high precision predictions of gene expression and promoter-enhancer interactions, including frequently interacting regions in HiC data, replication time, and 3D chromatin structure. AI can detect small amounts of biomarkers, significantly improve early cancer diagnosis and personalize clinical care while aiding in the discovery of new anticancer drugs. The maturation of epigenomic analysis provides synergistic opportunities for AI-based imaging efforts [150]. In general, the application of AI in precision cancer diagnosis and treatment presents broad prospects, resulting in the development of intelligent cancer treatments.

## Conclusion

The altered epigenome of tumor cells (including DNA methylation, histone tail modification, nucleosome localization, and abnormal patterns of 3D chromatin organization within the nucleus) are potentially effective biomarkers to detect cancer cells and classify tumor types. The development of various drugs targeting epigenetic modulators not only provide epigenetic-based therapies that are used to treat hematologic malignancies, but also demonstrate viable therapeutic potential for solid tumors in preclinical and clinical trials. Due to the limitations of epigenetic therapy in the treatment of solid tumors, potentially effective therapeutics for different malignancies (particularly combination strategies) deserve clinical consideration. Indeed, the cancer epigenome determines how cancer cells respond to therapeutic interventions; therefore, it is necessary to advance our understanding of the cancer epigenetics landscape as the clinical course of precision medicine is pursued. Advanced epigenetic therapy provides unique insights into various cancer treatment models that detect abnormal epigenetic changes. This represents an important step forward in the development of personalized precision diagnosis and malignant tumor treatment. New iterations of technology including (but not limited to) sequencing techniques and AI, and the continuous advancement of epigenetic therapies will open new avenues to establish precision diagnostics and therapeutics.

### Reporting summary

Further information on research design is available in the Nature Research Reporting Summary linked to this article.

### Supplementary information

Reporting Summary
